# Guideline “Transient Global Amnesia (TGA)” of the German Society of Neurology (Deutsche Gesellschaft für Neurologie): S1-guideline

**DOI:** 10.1186/s42466-023-00240-0

**Published:** 2023-04-20

**Authors:** Dirk Sander, Thorsten Bartsch, Florian Connolly, Christian Enzinger, Urs Fischer, Nils Nellessen, Holger Poppert, Kristina Szabo, Helge Topka

**Affiliations:** 1 Klinik für Neurologie, Neurologische Frührehabilitation und Weiterführende Rehabilitation, Benedictus Krankenhaus Tutzing und Feldafing, Bahnhofstraße 5, 82327 Tutzing, Germany; 2 Neurologische Universitätsklinik Schleswig-Holstein, Campus Kiel, Kiel, Germany; 3 Praxis für Neurologie, Hauptstraße 31–35, 14776 Brandenburg an der Havel, Germany; 4 Neurologische Universitätsklinik, Medizinische Universität Graz, Graz, Austria; 5 Neurologische Universitätsklinik, Universitätsspital Basel, Basel, Switzerland; 6 Klinik für Neurologie und Neurophysiologie, Helios Universitätsklinikum Wuppertal, Universität Witten-Herdecke, Wuppertal, Germany; 7 Neurologische Klinik, Helios Klinikum München West, Munich, Germany; 8 Neurologische Klinik, Universitätsmedizin Mannheim, Medizinische Fakultät der Universität Heidelberg, Mannheim, Germany; 9 Klinik für Neurologie, Neurophysiologie, Kognitive Neurologie und Stroke Unit, München Klinik Bogenhausen, Munich, Germany

**Keywords:** Amnesia, Transient, Memory impairment, Hippocampus, Differential diagnosis

## Abstract

**Introduction:**

In 2022 the DGN (Deutsche Gesellschaft für Neurologie) published an updated Transient Global Amnesia (TGA) guideline. TGA is characterized by a sudden onset of retrograde and anterograde amnesia for a period of one to a maximum of 24 h (with an average of 6 to 8 h). The incidence is estimated between 3 and 8 per 100,000 population/year. TGA is a disorder that occurs predominantly between 50 and 70 years.

**Recommendations:**

The diagnosis of TGA should be made clinically. In case of an atypical clinical presentation or suspicion of a possible differential diagnosis, further diagnostics should be performed immediately. The detection of typical unilateral or bilateral punctate DWI/T2 lesions in the hippocampus (especially the CA1 region) in a proportion of patients proves TGA. The sensitivity of MRI is considered higher when performed between 24 and 72 h after onset. If additional DWI changes occur outside the hippocampus, a vascular etiology should be considered, and prompt sonographic and cardiac diagnostics should be performed EEG may help to differentiate TGA from rare amnestic epileptic attacks, especially in recurrent amnestic attacks. TGA in patients < 50 years of age is a rarity, therefore it is mandatory to rapidly search for other causes in particular in younger patients. The cause of TGA is still unknown. Numerous findings in recent years point to a multifactorial genesis. Because the pathomechanism of TGA is not yet clearly known, no evidence-based therapeutic or prophylactic recommendations can be made.

**Conclusions:**

There is no evidence for chronic sequelae of TGA with respect to cerebral ischemia, chronic memory impairment, or the onset of dementia-related syndromes.

## Introduction

Transient global amnesia (TGA) is characterized by a sudden onset of retrograde and anterograde amnesia for a period of one to a maximum of 24 h with an incidence between 3 and 8 per 100,000 population per year. The diagnosis of TGA is based on the patient’s history and that of others, as well as on clinical neurological and orienting neuropsychological examination, in particular the exclusion of possible differential diagnoses. The diagnosis usually can be established in the acute stage based on the criteria of Caplan [1] and Hodges and Warlow [2]. In general, the prognosis of a TGA is very favorable. Since the pathomechanism of TGA has not yet been definitively clarified, a multifactorial or multi-causal process seems likely, As empirical data are also lacking, no sufficiently evidence-based recommendations can be made regarding prophylaxis.

This guideline is a translated version of the german guideline “Transiente globale Amnesie”. A complete version of this guideline (in German) can be found on the website of the Deutsche Gesellschaft für Neurologie (www.dgn.org/leitlinien) and the AWMF (Arbeitsgemeinschaft wissenschaftlicher Medizinischer Fachgesellschaften).

## Methods of guideline development

*AWMF Registry number:* 030-083.

*Level of guideline:* S1.

*Date of last update:* October 2022.

*Valid until:* 31.12.2025.

*Edited by:* Guidelines Committee of the German Neurological Society.

*Methodological approach:* The project was managed by the coordinator Dirk Sander. The topics were worked on by author teams based on the current data situation and then coordinated in several Delphi round by the guideline group. The guideline was reviewed by the Guideline Committee of the German Neurological Society and approved by the German Neurological Society.

*Joint recommendations:* German Neurological Society (DGN), Austrian Neurological Society (ÖGN) and Swiss Neurological Society (SNG).

## What’s new?

- Recent data show that the risk of TGA recurrence ranges from 12 to 27% [3–5].
- Patients with TGA are significantly more likely (OR 2.5) to have a history of migraine [6].
- The risk for TGA is higher in the morning (10–11 a.m.) and late afternoon (5–6 p.m.) [7].
- A meta-analysis of 36 studies found a significant association between TGA recurrence risk and migraine, depression, and sexual activity as trigger factors [5].
- Functional and morphometric MRI studies [8, 9] and quantitative EEG analyses [10] have demonstrated changes in functional connectivity in the limbic system and hippocampus.
- Up to 11% of patients with TGA additionally show small DWI changes outside the hippocampus [11].

## The most important recommendations

- The diagnosis of TGA should be made clinically. In case of an atypical clinical presentation or suspicion of a possible differential diagnosis, further diagnostics (imaging, preferably MRI, if necessary, EEG, laboratory, or CSF diagnostics) should be performed immediately.
- The diagnosis of TGA can be positively supported by MR imaging: The detection of typical unilateral or bilateral punctate DWI/T2 lesions in the hippocampus (especially the CA1 region) in a proportion of patients proves TGA. The sensitivity of MRI is considered higher when performed between 24 and 72 h after onset.
- Cranial MRI also helps to exclude other causes of the amnestic episodes, especially ischemic stroke If the presentation is atypical, immediate imaging is indicated.
- If additional DWI changes are outside the hippocampus, a vascular etiology should be considered, and prompt sonographic and cardiac diagnostics should be performed.
- TGA is a disorder that occurs predominantly between the ages of 50 and 70 years; TGA in patients < 50 years of age is a rarity, therefore it is mandatory to rapidly search for other causes in particular in younger patients.
- EEG can help differentiate from the rare amnestic epileptic attacks, especially in higher-frequency recurrent amnestic attacks (> 3/year). Absence of EEG abnormalities does not necessarily rule out an epileptic etiology.
- Inpatient monitoring can be considered for at least 24 h or until symptoms resolve, especially if there is no possibility of supervision by relatives.
- Because the pathomechanism of TGA is not yet clearly known, no evidence-based recommendations can be made regarding prophylaxis. Possible cerebrovascular risk factors should be identified and treated according to guidelines.
- To date, there is no evidence for the presence of chronic sequelae with respect to chronic memory impairment or the development of dementia-related syndromes. Also, there are no indications that TGA represents a risk factor for cerebral ischemia. Very subtle neuropsychological abnormalities may persist for longer periods of time but do not seem to render clinical significance in everyday life.

## Definition and clinical symptoms

Transient global amnesia (TGA) is characterized by a sudden onset of retrograde and anterograde amnesia for a period of one to a maximum of 24 h (with an average of 6 to 8 h). When presenting with typical clinical symptoms, even a rarely occurring episode duration < 1 h does not argue against the diagnosis of TGA [12]. During the episode, the retention span for new information is reduced to 30–180 s (anterograde amnesia). Affected individuals are therefore often not oriented with respect to time and situation but always oriented to person. There is no altered vigilance. Patients are awake and able to make contact. They appear perplexed and troubled and repeatedly ask questions about time and situational circumstances. In parallel, access to old memory acquired before TGA is often impaired (retrograde amnesia). Retrograde amnesia also leads to perplexity, as affected individuals cannot reconstruct the events of the preceding hours or days at all or only incompletely. During the acute episode, complex, previously learned activities such as driving a car, walking in familiar surroundings, or cooking can be performed.

After the TGA has subsided, a permanent amnestic gap of usually several hours, which covers the period of the acute interval, remains [13].

## Epidemiology and precipitating events

The incidence of TGA is estimated between 3 and 8 per 100,000 population per year. Most patients (75%) are between 50 and 70 years of age at onset. TGA in patients younger than 30 years has not been described.

In most patients (up to 85%), TGA is preceded by events that may be considered precipitating [14]. In some cases, TGA may occur spontaneously.

The risk of recurrence during the course ranges from 12 to 27% [3–5], with most patients (85%) experiencing 3 or fewer recurrences [3]. Predisposing factors for recurrence include the presence of migraine (odds ratio, OR 2.1), depression (OR 4.5), and sexual activity as a trigger factor (OR 1.5) [5]. There is a clustering of TGA in the morning (10–11 am) and late afternoon (5–6 pm) [7]. TGA occurs with approximately equal frequency in men and women.

## Pathophysiology

The cause of TGA is still unknown, but numerous findings in recent years point to a multifactorial genesis [15]. Based on the clinical picture, as a common pathophysiological correlate a dysfunction of mediobasal temporal lobe components including both hippocampi is assumed, as these structures are involved in both memory consolidation and retrieval [16]. This notion is supported by imaging findings using diffusion-weighted magnetic resonance imaging, which shows hippocampal lesions in more than half of patients in the acute stage [17–23].

TGA patients with and without DWI lesions do not differ in terms of clinical symptoms and cognitive functions over the long term [24–26]. Bartsch et al. [18] found no evidence of a persistent lesion in 20 TGA patients with initially existing hippocampal DWI lesions in the T2-weighted follow-up examination after 4–6 months. The number and volume of T2 hyperintense signals were comparable to those of healthy controls [27, 28].

Functional and morphometric MRI studies [8, 9] and quantitative EEG analyses [10] were able to show changes in functional connectivity in the limbic system and hippocampus. These changes were more clearly detectable in patients with recurrent TGA [9, 29].

Larner [30] developed a neural network model of memory function and suggested, among other things, that loss of fault tolerance of hippocampal neural networks leads to a failure of synaptic transmission in these networks and thus to memory impairment.

### Cerebral ischemia

Cerebral ischemia as a cause of TGA is considered unlikely. Several independent studies showed no consistent association with cardiovascular risk factors and no clustering of past or future cerebral infarcts in TGA patients [31–33]. Troponin C may be elevated in some TGA patients [34]. Eisele et al. [35] described asymptomatic troponin I elevation in 8.5% of their TGA patients.

Paradoxical cerebral emboli, favored by Valsalva maneuvers, triggering TGA has also been considered [36]. Although atrial septal defects seem to be much more frequent in TGA patients (55%) as compared to the general population (25%), it remains unexplained for the time being how paradoxical emboli could trigger such a monomorphic and always transient neurological deficit.

### Migraine equivalent

Based on some clinical parallels, a relationship between TGA and migraine has been suggested by several authors [37, 38]. Interestingly, 12–30% of TGA patients have a history of migraine [37, 39]. Approximately 10% of TGA patients experience headaches during or immediately after the attack. Several case–control studies found an increased prevalence of migraine in TGA patients. In a recent meta-analysis of 12 studies, patients with TGA were significantly more likely (OR 2.5; 95% CI 1.85–3.41) to have a history of migraine [6].

Arguing against an interpretation of TGA as a migraine equivalent is the fact that as many as 23% of all people experience at least one classic migraine attack in their lifetime [40] which contradicts the significantly lower prevalence of TGA. Also, migraine decreases in severity and frequency with age, whereas TGA reaches its frequency peak at older ages.

### Venous congestion

Various authors [36, 41, 42] have pointed out the frequency of pre-TGA situations involving a Valsalva-like maneuver. A summary [14] of 12 TGA studies involving 837 patients provided further evidence of a preceding Valsalva-like maneuver (in descending frequency: physical activity, sexual intercourse, swimming) in 44% (26–85%) of TGA patients. Lewis [43] suggested that there might be reduced venous return to the heart and concomitant intracranial venous hypertension due to the increased intrathoracic pressure that occurs during such Valsalva-like maneuvers. This mechanism could be associated with consecutive passive venous ischemia of memory-relevant areas [44]. Duplex sonographic studies [45–51] found incompetent internal jugular vein valves significantly more often in TGA patients than in control subjects (73% vs. 31%; *p* < 0.0001), which could favor venous congestion. However, Kang et al. [52] did not demonstrate more frequent intracranial jugular venous reflux in TGA patients compared with controls using “time-of-flight” MRA examinations. Han et al. [53], on the other hand, demonstrated in a combined study using ultrasound and contrast enhanced MRV that a Valsalva maneuver leads to a reduction in venous flow in the jugular veins in TGA patients compared with healthy controls. In general, however, the very low rate of recurrence of TGA seems difficult to be concealed with persistent structural lesions of the cranial venous system.

### Mental and physical stressors

TGA is often preceded by an emotionally stressful event or physical exertion [54]. TGA patients are significantly more likely to have phobic or anxious personality traits [15] and significantly more likely to have a history of psychiatric disorders as compared to patients with TIA (39.2% vs. 13.7%) [55]. A study by Döhring et al. [56] concluded that TGA patients may cope with stress less effectively and constructively. Furthermore, evidence exists for greater cortisol secretion and stress-related hyperreactivity in TGA patients [57].

In summary, these findings most likely suggest that TGA is based on a transient dysfunction, particularly of the hippocampus, based on a multifactorial etiology, such that several of the factors described above must interact for TGA to occur.

## Prognosis

In general, the prognosis of a TGA is very favorable: Although the acute amnesia usually disappears within 24 h, some patients sometimes still feel subjectively impaired and irritated for several days after the attack. Population-based studies and meta-analyses also showed complete regression of symptoms over the long-term course [58–61].

## Diagnostics

The diagnosis of TGA is based on the patient’s history and that of others, as well as on the neurological and bedside neuropsychological examination and the exclusion of possible differential diagnoses (see below). The diagnosis usually can be established primarily clinically in the acute stage based on the criteria of Caplan [1] and Hodges and Warlow [2]:

- Acute onset and pronounced new memory impairment.
- Duration of at least 1 h, regression within 24 h.
- No focal neurological symptoms/deficits and no additional cognitive deficits.
- Absence of impaired consciousness or disorientation to person.
- No previous trauma or epilepsy.

Clinical symptoms beyond isolated memory impairment with antero- and retrograde amnesia and mild vegetative symptoms, i.e., somnolence, severe headache, vomiting, confusion, fever, etc., or incomplete recovery after more than 24 h argue against TGA and require rapid differential workup to rule out a potentially dangerous neurologic disorder.

### Practical guidelines for the diagnosis of acute TGA [13]

- Previous strenuous physical or emotional events.
- Selective memory loss (e.g., 3-word test/word list, retrograde memory).
- The patient repeatedly asks the same questions.
- The patient is cooperative and can name things.

### Speaking against a TGA

- Evidence of metabolic disturbances (including electrolyte disturbance), trauma, or known epilepsy.
- Other neurologic symptoms or focal neurologic deficits.
- Quantitative or qualitative disorders of consciousness, and other cognitive disorders.
- The patient can describe details and timing of acute amnestic interval.
- The patient exhibits retrograde amnesia alone.
- Evidence of more frequent amnestic episodes (> 3/year).

## Recommended diagnostic procedures

In case of factors that clearly point against TGA or presence of persistent symptoms, preferably inpatient monitoring should be performed to evaluate the further course as well as emergency cerebral imaging (preferably MRI). If symptoms have already resolved, further inpatient treatment is necessary only for findings that cast doubt on TGA.

On MRI, typical DWI lesions are most likely to appear 24–72 h after TGA, especially in the CA1 region (about 30% of lesions) [62] of the hippocampus, most of which are accompanied by T2 hyperintensity and are still detectable 10–14 days after TGA [23]. Detection of these DWI lesions support the diagnosis of TGA and is found in up to ¾ of all patients [21]. However, the absence of DWI lesions does not exclude TGA.

Up to 11% of patients with typical TGA also have small DWI changes outside the hippocampus [11, 63]. This finding should prompt an expanded embolic source search. To date, there is no evidence of residual structural lesions secondary to DWI lesions.

Using “diffusion tensor imaging”, it has been shown that patients with TGA recurrence do not have detectable structural damage to memory network fibers, which has been postulated as a possible predisposition for recurrence [64]. TGA patients also do not show increased vascular risk and increased risk of stroke [32, 33].

### Practical recommendations for imaging TGA patients include [19, 21, 64]

- MRI (preferably 3 T); the best visualization of possible DWI lesions is achieved in the time window of 24–72 h after TGA.
- DWI/ADC/T2 sequences should be performed.
- Angulation axial and coronal correspond to the longitudinal axis of the hippocampus.
- Low slice thickness of 3 mm (DWI) or 2 mm (T2) to minimize partial volume effects.
- A high B value (b = 2000–3000 s/mm^2^) improves the detection of DWI lesions.
- Cerebral imaging at 24–72 h after onset seems best to confirm the tentative diagnosis of TGA. However, if the clinical presentation is atypical, cerebral imaging should be performed on an emergency basis to avoid missing a potentially treatable relevant condition (i.e., cerebral ischemia, encephalitis, etc.).

### Diagnostics required in individual cases

#### EEG

Affected individuals have an inconspicuous or only nonspecifically altered EEG; occasionally, theta and delta waves are found in the temporal leads. EEG may facilitate differentiation from amnestic epileptic attacks [65–67].

#### Cerebrovascular diagnosis

To date, it has not been convincingly demonstrated that—according to the strict diagnostic criteria of Caplan [1]—TGA represents a consequence of arterio-arterial or cardiac embolism. Also, patients do not have an increased risk of stroke after TGA [33].

#### Neuropsychological testing

Differentiated neuropsychological test batteries can detect subtle impairments in cognitive performance days to months after the event, contrary to the actual TGA definition, which calls for complete restitution within 24 h [68–70], although permanent impairment of memory functions was not detectable more than 3 years after TGA [58, 71].

### No indication

99mTc-SPECT or PET.

## Differential diagnosis

With an atypical clinical presentation of acute amnesia and/or accompanying focal neurologic deficits, ischemic stroke (PCA infarction involving the hippocampus) must be excluded by MRI.

Essentially, TGA must be clinically differentiated from transient epileptic amnesia (TEA) [66, 67, 72]. Affected individuals are also able to perform complex activities (e.g., writing, telephoning) and ask repetitive, anxious questions [73]. However, the interictal EEG is usually abnormal, and there is often evidence of classic complex-partial seizures in history.

The diagnostic criteria according to Zeman et al. [66] and Butler et al. [67] require, in addition to higher frequencies (> 3–5/year) amnestic intervals compared to TGA, evidence of the presence of epilepsy through the demonstration of epileptiform abnormalities in the EEG, other clinical signs of epilepsy and the prompt effect of anticonvulsant medication. TGA and TEA can also occur sequentially, with hippocampal damage in the setting of TGA possibly representing an epileptic focus of TEA [73].

Other differential diagnoses (e.g., Craniocerebral trauma, Intoxication, Hypoglycemia, etc.) lead to acute-onset memory impairment that can usually be readily delineated clinically and by history.

## Prophylaxis

Between 12 and 27% of affected individuals [3–5] suffer at least one additional TGA subsequently, which is perceived as dramatic by both patients and relatives despite the benign nature of the syndrome. Since the pathomechanism of TGA has not yet been definitively clarified, it is probably a multifactorial or multi-causal process, and empirical data are also lacking, no sufficiently evidence-based recommendations can be made regarding prophylaxis. Also, given the low rates of recurrence, prophylactic treatment may not be necessary.

## Data Availability

Not applicable.
