# Correction: Guideline “Transient Global Amnesia (TGA)” of the German Society of Neurology (Deutsche Gesellschaft für Neurologie): S1-guideline

**DOI:** 10.1186/s42466-023-00296-y

**Published:** 2023-11-08

**Authors:** Dirk Sander, Thorsten Bartsch, Florian Connolly, Christian Enzinger, Urs Fischer, Nils Nellessen, Holger Poppert, Kristina Szabo, Helge Topka

**Affiliations:** 1 Klinik für Neurologie, Neurologische Frührehabilitation und Weiterführende Rehabilitation, Benedictus Krankenhaus Tutzing und Feldafing, Bahnhofstraße 5, 82327 Tutzing, Germany; 2 Neurologische Universitätsklinik Schleswig-Holstein, Campus Kiel, Kiel, Germany; 3 Praxis für Neurologie, Hauptstraße 31?35, 14776 Brandenburg an der Havel, Germany; 4 Neurologische Universitätsklinik, Medizinische Universität Graz, Graz, Austria; 5 Neurologische Universitätsklinik, Universitätsspital Basel, Basel, Switzerland; 6 Klinik für Neurologie und Neurophysiologie, Helios Universitätsklinikum Wuppertal, Universität Witten-Herdecke, Wuppertal, Germany; 7 Neurologische Klinik, Helios Klinikum München West, Munich, Germany; 8 Neurologische Klinik, Universitätsmedizin Mannheim, Medizinische Fakultät der Universität Heidelberg, Mannheim, Germany; 9 Klinik für Neurologie, Neurophysiologie, Kognitive Neurologie und Stroke Unit, München Klinik Bogenhausen, Munich, Germany

**Correction: Neurol. Res. Pract. 5, 15 (2023)**

10.1186/s42466-023-00240-0

Following publication of the original article [1], the authors reported that the author name “Nils Nellessen” was incorrectly written as “Nils Nellesen”.

The original article [1] has been updated.

## References

[1] Sander, D., Bartsch, T., Connolly, F. et al (2023). Guideline “Transient Global Amnesia (TGA)” of the German Society of Neurology (Deutsche Gesellschaft für Neurologie): S1-guideline. *Neurol. Res. Pract.* 5, 15 . 10.1186/s42466-023-00240-0 PMC10116751 37076927

